# Treatment Contact Coverage for Probable Depressive and Probable Alcohol Use Disorders in Four Low- and Middle-Income Country Districts: The PRIME Cross-Sectional Community Surveys

**DOI:** 10.1371/journal.pone.0162038

**Published:** 2016-09-15

**Authors:** Sujit D. Rathod, Mary J. De Silva, Joshua Ssebunnya, Erica Breuer, Vaibhav Murhar, Nagendra P. Luitel, Girmay Medhin, Fred Kigozi, Rahul Shidhaye, Abebaw Fekadu, Mark Jordans, Vikram Patel, Mark Tomlinson, Crick Lund

**Affiliations:** 1 Centre for Global Mental Health, Department of Population Health, London School of Hygiene and Tropical Medicine, London, United Kingdom; 2 Butabika National Referral and Teaching Hospital, Makerere University, Kampala, Uganda; 3 Alan J. Flisher Centre for Public Mental Health, Department of Psychiatry and Mental Health, University of Cape Town, Cape Town, South Africa; 4 Sangath, Bhopal, India; 5 Transcultural Psychosocial Organization (TPO) Nepal, Kathmandu, Nepal; 6 Aklilu Lemma Institute of Pathobiology, Addis Ababa University, Addis Ababa, Ethiopia; 7 Centre for Mental Health, Public Health Foundation of India, New Delhi, India; 8 CAPHRI School for Public Health and Primary Care, Maastricht University, Maastricht, Netherlands; 9 Department of Psychiatry, College of Health Sciences, Addis Ababa University, Addis Ababa, Ethiopia; 10 Centre for Affective Disorders and the Affective Disorders Research Group, Institute of Psychiatry, Psychology and Neuroscience, King's College London, London, United Kingdom; 11 Research and Development Department, HealthNet TPO-Amsterdam, Amsterdam, Netherlands; 12 Centre for Global Mental Health, Institute of Psychiatry, Psychology and Neuroscience, King’s College London, London, United Kingdom; 13 Alan J. Flisher Centre for Public Mental Health, Department of Psychology, Stellenbosch University, Stellenbosch, South Africa; Medizinische Universitat Wien, AUSTRIA

## Abstract

**Context:**

A robust evidence base is now emerging that indicates that treatment for depression and alcohol use disorders (AUD) delivered in low and middle-income countries (LMIC) can be effective. However, the coverage of services for these conditions in most LMIC settings remains unknown.

**Objective:**

To describe the methods of a repeat cross-sectional survey to determine changes in treatment contact coverage for probable depression and for probable AUD in four LMIC districts, and to present the baseline findings regarding treatment contact coverage.

**Methods:**

Population-based cross-sectional surveys with structured questionnaires, which included validated screening tools to identify probable cases. We defined contact coverage as being the proportion of cases who sought professional help in the past 12 months.

**Setting:**

Sodo District, Ethiopia; Sehore District, India; Chitwan District, Nepal; and Kamuli District, Uganda

**Participants:**

8036 adults residing in these districts between May 2013 and May 2014

**Main Outcome Measures:**

Treatment contact coverage was defined as having sought care from a specialist, generalist, or other health care provider for symptoms related to depression or AUD.

**Results:**

The proportion of adults who screened positive for depression over the past 12 months ranged from 11.2% in Nepal to 29.7% in India and treatment contact coverage over the past 12 months ranged between 8.1% in Nepal to 23.5% in India. In Ethiopia, lifetime contact coverage for probable depression was 23.7%. The proportion of adults who screened positive for AUD over the past 12 months ranged from 1.7% in Uganda to 13.9% in Ethiopia and treatment contact coverage over the past 12 months ranged from 2.8% in India to 5.1% in Nepal. In Ethiopia, lifetime contact coverage for probable AUD was 13.1%.

**Conclusions:**

Our findings are consistent with and contribute to the limited evidence base which indicates low treatment contact coverage for depression and for AUD in LMIC. The planned follow up surveys will be used to estimate the change in contact coverage coinciding with the implementation of district-level mental health care plans.

## Background

Mental, neurological and substance use disorders are the fifth-leading cause of disability-adjusted life years [1], with up to 3.8% of the global burden attributed to depression [2] and 4.6% to alcohol consumption [3]. A robust evidence base is now emerging that indicates that treatment for depression [4] and for alcohol use disorders (AUD) [5] delivered in low and middle-income countries (LMIC) can be effective and cost-effective. These developments have contributed to the elevation of mental health on the global agenda, most notably evidenced through the United Nations Sustainable Development Goals 3.4 and 3.5 [6].

A key indicator for monitoring these two development goals is contact coverage [7], which is defined as the proportion of affected individuals who seek help with a service provider [8]. The only prior effort to estimate the coverage in multiple countries using a common methodology was the World Mental Health Surveys [9,10], which reported a mental health treatment gap of over 75% for serious cases residing in LMIC. The WHMS found substantial cross-national variation in its results [7]; this variation calls into question the assumption that the burden of mental illness (which is the denominator for contact coverage) in a country or region of countries can be imputed from its neighbours, as has been done extensively in the Global Burden of Disease Study [1]. The WMHS also surveyed a limited number of LMIC. As such, there is acute need for additional primary data collection on the burden of mental illness and of treatment coverage in LMIC.

The purpose of this population-based multi-round cross-sectional study is to estimate the change in treatment contact coverage as a result of implementing a district level mental healthcare plan for adult residents who are affected by depression or by alcohol use disorders in selected districts in Ethiopia, India, Nepal and Uganda. The aims of this paper are to describe the methods of the study and to report the baseline findings regarding treatment contact coverage for depression and for AUD in the 4 district sites.

## Materials and Methods

### Context, setting and participants

The PRogramme for Improving Mental health carE (PRIME) Research Programme Consortium aims to reduce the population-level burden of mental illness by implementing and evaluating district mental health care plans (MHCPs) in Ethiopia, India, Nepal, South Africa and Uganda [11]. In common with findings from many LMIC, the populations in the PRIME countries are characterised by high burden of mental disorders, limited awareness of the nature and treatability of mental illness, and weak health system capacity for diagnosis, treatment and rehabilitation services [12,13]. In collaboration with the respective Ministries of Health, PRIME consortium partners are integrating detection and treatment for depression, alcohol use disorder, psychosis and epilepsy into the primary health care setting [11,14]. In consultation with the Ministries of Health in each country, district sites were selected as suitable for demonstration of proof-of-concept for integration, and could provide evidence for potential future national scale up [14]. Detailed descriptions of the processes used to develop the overall evaluation methodology for PRIME are available [15,16].

Our population of interest consists of adult residents of four of the five PRIME implementation areas, (i.e. Sodo District, Ethiopia; Sehore District, India; 10 Village Development Committees in central Chitwan District, Nepal; and Kamuli District, Uganda). We did not conduct the community survey in South Africa, as the mental health services there targeted chronic care patients within the primary healthcare system, and not the general population. We have defined treatment contact coverage using the framework described by Tanahashi [8], which is the proportion of individuals with a health condition who accessed a health care provider for that condition in the past 12 months. We identified affected individuals using screening tools, and reported the proportion of screen positive individuals in the population.

The eligibility criteria for participating in the community survey were: being above 18 years of age, residency in the implementation area, fluency in the dominant language in that area, and willingness to provide informed consent.

### Sample size

The sample sizes for each site’s survey were set to allow detection of a change in contact coverage between the baseline round and a planned endline round for each disorder, with 80% statistical power and a two-sided alpha of 0.05. For the effect size the country research teams and Ministry of Health officials hypothesized that the contact coverage would be between 0 and 5% at baseline, which would increase to between 20 and 30% at endline. Other required parameters were the expected prevalence of the mental disorders, and the sensitivities and specificities of the screening tools, namely the Alcohol Use Disorders Identification Test (AUDIT) [17,18] for AUD and the Patient Health Questionnaire (PHQ-9) [19] for depression. For country teams opting to use a cluster sampling design, we also incorporated into the sample size calculation an intraclass correlation of 0.1 [20], and the number of clusters the field team could visit in the data collection period.

The sampling design specifications and data collection periods for each of the four implementation areas are described in Table 1.

**Table 1:** Sampling design for the baseline PRIME Community Survey, 2013–2014.

Implementation area	Sodo District, Ethiopia	Sehore District, India	Chitwan District*, Nepal	Kamuli District, Uganda
**Population size**	143,507 (total) [21]	1,336,235 [22] (total)	69,068 [23] (adults)	490,255 [24] (total)
**Baseline survey dates**	March-April 2014	May-June 2013, January-March 2014	May-August 2013	May-July 2013
**Final sample size (# adults)**	1,486	3,220	2,040	1,290
**Stage 1 sampling**	SRS of 1556 households from a district-level census list	PPS sample of 89 villages, proportionally allocated by 2 strata (urban/rural)	SRS of households, proportionally allocated by 90 strata (wards) in 10 Village Development Committees	PPS sample of 30 villages in the district
**Stage 2 sampling**	SRS of 1 adult within each household	SRS of 1 voting list per village	SRS of 1 adult within each household; any other perinatal women in the household were purposively selected.	SRS of 24–60 households in the village
**Stage 3 sampling**	Not applicable	Systematic sample of 25–47 adults from each voting list	Not applicable	SRS of 1 available adult in the household
**Replacement units in sample**	52 households	0 villages, 1937 adults	172 households	1 village, 3 households
**Individuals providing informed consent (%)**	1486/1508 (98.5)	3220 / 3233 (99.6%)	2040/2063 (98.9)	1290/1291 (99.9)

PPS = probability proportional to size, SRS = simple random sample.

*The implementation area includes 10 of the 36 Village Development Committees in Chitwan District.

### Sampling and field work

Characteristics of each of the districts sites and the rationale for their selection have been described previously [13]. In Nepal, the total sample size was distributed in proportion to the population size of the 90 wards in the 10 Village Development Committee (VDCs) which comprised the PRIME implementation areas of Chitwan District; information about ward size was provided by the respective VDC offices. Within each ward, field workers enumerated and listed all households and used a random procedure to select the required number of households from the list.

In Uganda, 30 villages in the district were randomly selected with probability being proportionate to size (PPS), with information about the village populations coming from Uganda 2002 census data. Within the selected village, field workers identified the central point in the village, spun a bottle to randomly pick a direction, selected households at a fixed sampling interval until reaching the village boundary, returned to the village centre and repeated the exercise until the target sample was reached. While the target sample per village was fixed at 60, in some cases the actual sample size was as a low as 25 due to constraints on the length of time field workers could remain in that village during working hours.

In Ethiopia, health extension workers completed a census of households in Sodo District between October and December 2013. They selected households from their census list with simple random sampling.

At the household level, the procedures used in Nepal, Uganda and Ethiopia are essentially identical. If no one was found at the household after three attempts to visit it, or no one was willing to provide a roster of residents, or the selected adult was not willing to provide informed consent, the fieldworker would select the closest neighbouring household. The field worker made three attempts to complete a scheduled interview before selecting another adult from the closest neighbouring household. At a selected household, the fieldworkers asked any household resident to provide the names and ages of all residents, and then used a random selection procedure to select one adult.

In India, data were collected in two waves. In Wave 1 (May-June 2013), 44 clusters (villages in rural areas, wards in urban areas) were selected in Sehore District using PPS, stratified by urban/rural areas. Information about the cluster’s population size was drawn from India 2001 census data. Within each cluster, fieldworkers enumerated and randomly selected one polling station, then downloaded the polling station’s list of registered voters from the electoral commission website. The fieldworkers sorted the list by gender and age, systematically selected voters using a fixed sampling interval, and attempted to schedule those voters for an interview. If it was not possible to contact the adult or the adult was not willing to provide informed consent, then the field workers selected the next adult named on the cluster’s voting list. Given constraints on travel time to remote village areas, the field workers could, in practice, interview between 25 and 47 adults in each village, totalling 1365 total participants. After the conclusion of Wave 1, the investigators and Ministry officials agreed to re-focus the implementation on one sub-district in Sehore District. In order to maintain statistical power to detect a change in contact coverage for this sub-district, we decided to conduct a second wave of the survey between January and March 2014. The procedures for Wave 2 were the same as Wave 1, but for data collection only in the single sub-district and using India 2011 census data, which had become available in the interim period. In Wave 2, 45 clusters were selected and another 1855 participant enrolled. Data from both waves are combined and presented here.

In all countries the field worker provided information about the survey in oral and written format to the selected adult. The selected adult then gave informed consent form with a signature or a thumbprint. The field worker started the questionnaire in a location selected by the participant. The field workers were residents of the areas in and around the implementation districts, had completed secondary education and were fluent in the local language. Interviews in all implementation sites were conducted between May 2013 and April 2014.

### Study measures

Interviewers orally administered a standard questionnaire consisting of two parts; the sections in Part 1 and Part 2 are detailed in Table 2. Part 1 was administered to all participants, and included sections to identify those who had probable depression or probable alcohol use disorders. Participants who scored ≥20 on the AUDIT were considered to have dependent alcohol use disorder, those who score 16–19 had harmful drinking, and 8–15 had hazardous drinking, with these categories defined per International Classifications of Disease version 10 (ICD-10) criteria [25]. Participants who had screening scores of 8 or more were considered to have probable alcohol use disorder, as suggested by the World Health Organization [18]. On the PHQ-9, a score of 10 or more was considered positive for depressive disorder, which is a commonly-used cut-off score [26]. The cut off points were set by considering validation studies of the respective screening instruments [26,27] to find the appropriate balance between maximizing the sensitivity (to reach a greater number of affected individuals) and maximizing the hypothesized positive predictive value (to minimize false positives). Immediately after completing the PHQ-9, each participant was asked “Apart from these past two weeks, during the past 12 months, did you have other episodes of two weeks or more when you felt depressed or uninterested in most things, and had most of the problems we just talked about?” The response to this question allows for assessment of depressive episodes in a 12-month recall period; for the purpose of this evaluation we considered participants who provided affirmative response to this question or who screened positive to have probable depressive disorder. In Nepal, after reaching a target sample of 1500 participants, in response to finding unexpectedly few adults who screened positive on the PHQ9, the research team decided to use an alternative translation for the term “mental health” and collect data on another 500 participants in a second wave of data collection.

**Table 2:** Questionnaire sections for the PRIME Community Survey.

Questionnaire section	Number of items	Source
**PART 1**		
**Basic demographic characteristics**	4	Purpose-built for the PRIME questionnaire
**Depression screening**	9	Patient’s Health Questionnaire (PHQ-9) [19]
**Recent history of depression-related symptoms**	1	Adapted from Mini International Neuropsychiatric Interview (MINI) [28] item A4a
**Treatment provision for depression-related symptoms^a^**	20	Purpose-built for the PRIME questionnaire
**Alcohol use disorder screening**	10	Alcohol use disorder identification test (AUDIT) [18]
**Treatment provision for problems with drinking^b^**	22	Purpose-built for the PRIME questionnaire
**Suicidal ideation and action**	8	Adapted from the Composite International Diagnostic Interview (CIDI) [29] suicidality module
**PART 2^a^^b^^c^**		
**Detailed demographic characteristics**	7	Purpose-built for the PRIME questionnaire
**Mental health-related knowledge, attitudes and behaviours**	17	Selected questions drawn from Mental Health Knowledge Schedule (MAKS) [30], Reported and Intended Behaviour Scale (RIBS) [31] and Community Attitudes to the Mentally Ill (CAMI) [32]
**Disability**	24	WHO Disability Assessment Schedule (WHODAS 2.0) 12-item and WHODAS 2.0 caregiver questions [33]
**Inpatient care services received**	6	Selected questions from Client Service Receipt Inventory (CSRI) [34]. Four questions repeat per inpatient visit.
**Outpatient health care services received**	16	Selected questions from CSRI [34]. Eleven questions repeat per outpatient visit.

^a^ For participants who screen positive for depression.

^b^ For participants who screen positive for AUD.

^c^ For selected participants who had negative screening results for depression and for AUD.

Immediately after each screening section, for participants who were considered to have the disorder in question, interviewers in India, Nepal and Uganda asked whether they had sought treatment for that disorder over the past 12 months. Interviewers in Ethiopia used different cut off scores (PHQ9 score of 7 or more, AUDIT score of 16 or more, which reflected projected capacity for treatment provision at these scores) and asked whether the participant had sought treatment at any time in their life. A participant who responded in the affirmative was considered to have contact coverage for the disorder. Next, the interviewer asked “From whom did you receive treatment?” Responses were grouped into the following three categories: Specialist mental health provider (psychiatrist, psychologist, counsellor, mental health nurse), generalist health provider (medical doctor, clinical officer, social worker, community health worker, nurse), or complementary provider (traditional healer, religious or spiritual advisor, other provider). These three provider groupings were used in a survey of mental health service use conducted by Williams et al in South Africa [35]. In India, Nepal and Uganda the participant could list multiple providers, and in Ethiopia the participant was asked to name the first provider from whom they ever sought treatment. For each provider category, we asked follow up questions about the nature of treatment (i.e. medication, counselling, other), how much those treatments helped, and about satisfaction with the providers.

Part 2 of the questionnaire was administered to all participants in India, and to all participants who screened positive for depression or AUD in the remaining countries. In addition, a random 10% subsample of participants who screened negative on both selections in Nepal completed Part 2.

The sections of the cross-country questionnaire which were developed specifically for PRIME are available on request.

### Data collection

In India and Nepal the field workers collected data on Android tablets with a questionnaire application [36]. These field workers administered Hindi and Nepali versions of the questionnaire, respectively, with questions and response options displayed in Devanagari script. In Ethiopia and Uganda, the field workers collected data with pencil and paper, and administered the questionnaire in Amharic and Luganda, respectively. In Ethiopia data entry operators double entered paper questionnaire responses into an Epidata [37] database, while in Uganda, a data entry operator single entered paper questionnaire responses into an English-language version of the same tablet questionnaire application used in India and Nepal. The application automatically transmitted completed questionnaire data via mobile or wireless network to a secure server housed by the application provider, and the data was then automatically deleted from the device.

### Analysis

First, we describe the demographic characteristics of adults in PRIME implementation areas. Second, we calculate the reliability of the PHQ9 and AUDIT tools in each county with Cronbach’s alpha. Next, for each implementation site, we report the proportion of adults who screen positive on the PHQ9 and who have a recent history of depressive symptoms, and who screen positive on the AUDIT. For adults who have probable depression or probable alcohol use disorders, we report the proportion who contacted a specialist, generalist or complementary health provider. We present the screening and contact coverage figures for the total adult population, and also stratified by sex.

For continuous measurements, we present the means and standard deviations. For categorical measurements, we presented percentages. All figures are adjusted for complex sampling designs. The analysis was conducted in Stata 13.1. [38] Stata code in S1 Analysis.

### Ethics

The institutional review boards of the World Health Organization (Geneva, Switzerland), University of Cape Town (South Africa), Addis Ababa University (Ethiopia), Indian Council of Medical Research (New Delhi, India), Sangath (Goa, India), Nepal Health Research Council (Kathmandu, Nepal), Makerere University (Kampala, Uganda), and the National Council of Science and Technology (Kampala, Uganda) reviewed and approved the protocol for the PRIME Community Survey.

## Results

We reached or exceeded the target sample sizes at each study site, which ranged from 1290 in Uganda to 2040 in Nepal. In all settings, there was minimal replacement of sampling units (e.g. villages or households) and large majorities of eligible adults who were invited to participate (all >98%) provided informed consent (Table 1). The demographic characteristics of adults in PRIME implementation areas are described in Table 3. The mean age of adults ranged between 36.0 years in Uganda to 40.2 years in India. There was more variation in the proportion of female participants, from 45.4% in India to 65.6% in Uganda. Educational attainment was also varied: the majority of adults had not completed primary education in India (50.9%), in Uganda a majority (52.6%) completed primary education but not secondary, and in Nepal a majority (54.5%) completed secondary education.

**Table 3:** Demographic characteristics of adults in PRIME implementation areas, 2013–2014.

Characteristic (mean and SD, or %)		Sodo District, Ethiopia	Sehore District, India	Chitwan District, Nepal	Kamuli District, Uganda
**Age (SD), years**		39.4 (15.3)	40.2 (15.1)	39.8 (15.2)	36.0 (13.8)
**Female sex (%)**		50.6	45.4	59.8	65.6
**Religion (%)**					
	Muslim	3.0	10.2	<0.1	*
	Hindu	0.0	89.8	80.8	*
	Buddhist	0.0	0.0	17.1	*
	Christian	96.9	0.0	2.0	*
	Other	0.1	<0.1	<0.1	*
**Educational status (%)**					
	Less than primary education / illiterate	45.6	50.9	12.8	16.9
	Completed primary education / literate	46.0	36.6	32.7	52.6
	Completed secondary education	8.4	12.6	54.5	30.5

*Population-level estimate not available; only measured for participants who screened positive for depression or alcohol use disorders.

For the PHQ9 the Cronbach’s alphas in the countries were all above 0.79 (Ethiopia 0.81, India 0.79, Nepal 0.79, Uganda 0.79) and for the AUDIT the alphas were all above 0.71 (Ethiopia 0.84, India 0.87, Nepal 0.77, Uganda 0.71). The screening results and treatment contact coverage figures are described in Tables 4 and 5. The percentage of adults who screened positive for depression on the PHQ-9 ranged between 3.6% in Nepal to 17.7% in India. In Nepal, adults were less likely (9.6%) to agree that they had experienced symptoms consistent with depression for another period of two weeks in the previous year than at the other three sites, where nearly one in four adults did agree. Of the adults who did have symptoms consistent with current or recent depression, treatment contact coverage over the past 12 months ranged between 8.1% in Nepal to 23.7% in Uganda. In Ethiopia, lifetime contact coverage for probable depression was 23.9%.

**Table 4:** Treatment contact coverage for probable depressive disorder in PRIME implementation areas, 2013–2014.

Characteristic (%, and 95% CI)		Sodo District, Ethiopia			Sehore District, India			Chitwan District, Nepal			Kamuli District, Uganda		
		Total	Men	Women	Total	Men	Women	Total	Men	Women	Total	Men	Women
**PHQ9 positive (%)**		8.0^b^ (6.6–9.6)	5.9^b^ (4.2–8.2)	9.9^b^ (7.9–12.5)	17.7 (16.3–19.1)	16.8 (15.1–18.6)	18.7 (16.8–20.8)	3.6 (2.8–4.6)	3.6 (2.3–5.5)	3.6 (2.7–4.7)	6.5 (4.3–9.7)	4.3 (2.7–6.7)	7.8 (4.8–12.7)
**Recent (12 months) history of depressive symptoms (%)**		24.1 (21.8–26.6)	22.9 (19.7–26.5)	25.2 (22.0–28.7)	23.5 (21.7–25.5)	20.7 (18.5–23.2)	26.9 (24.2–29.9)	9.6 (8.2–11.3)	8.1 (6.0–10.8)	10.7 (8.7–13.0)	22.7 (19.0–26.8)	19.9 (15.4–25.3)	26.3 (21.7–31.5)
**PHQ9-positive or recent history of depressive symptoms (%)**		25.3^b^ (23.0–27.8)	23.4^b^ (20.1–27.0)	27.2^b^ (24.0–30.7)	29.7 (27.9–31.6)	26.3 (24.2–28.7)	33.9 (31.0–36.7)	11.2 (9.7–13.0)	10.0 (7.7–12.8)	12.1 (10.1–14.4)	23.2 (19.5–27.4)	20.7 (16.1–26.0)	26.8 (22.1–31.9)
**Treatment contact coverage (of those who are PHQ9 positive or have recent history of depressive symptoms) (%)**		23.9^a^^b^ (19.8–28.6)	24.9^a^^b^ (18.8–32.4)	23.1^a^^b^ (17.9–29.3)	12.8 (10.5–15.6)	12.3 (9.5–15.9)	13.1 (9.8–17.3)	8.1 (4.6–13.3)	6.3 (2.6–14.5)	9.1 (4.6–17.0)	23.7 (17.7–31.1)	22.5 (13.2–35.6)	26.9 (20.6–34.3)
	Contact coverage from specialist health provider (%)	0.2^a^^b^ (0.0–1.3)	0.4^a^^b^ (0.05–2.9)	0.0	3.0 (1.9–4.7)	4.4 (2.6–7.2)	1.8 (0.8–3.7)	3.5 (1.7–6.8)	4.5 (1.7–11.5)	2.9 (1.2–7.0)	2.4 (1.1–5.2)	4.1 (1.3–12.5)	0.2 (0.03–1.6)
	Contact coverage from generalist health provider (%)	12.2^a^^b^ (9.2–16.2)	11.2^a^^b^ (7.0–17.5)	13.1^a^^b^ (9.0–18.6)	7.8 (6.0–10.2)	6.6 (4.5–9.6)	9.0 (6.5–12.4)	1.7 (0.6–4.6)	1.8 (0.3–12.0)	1.6 (0.6–4.6)	16.8 (12.4–22.3)	14.6 (8.5–23.9)	20.7 (15.0–27.9)
	Contact coverage from complementary provider (%)	10.5^a^^b^ (7.8–13.9)	11.2^a^^b^ (7.2–17.1)	9.9^a^^b^ (6.6–14.4)	2.6 (1.8–3.8)	1.9 (1.0–3.6)	3.4 (2.1–5.3)	4.0 (1.6–9.4)	0.5 (0.07–3.7)	6.0 (2.3–14.5)	3.9 (1.8–8.6)	3.8 (0.8–16.3)	4.0 (2.0–7.7)

^a^ Lifetime treatment contact coverage, rather than over the past 12 months.

^b^ Using a PHQ-9 cut off score of ≥7.

**Table 5:** Treatment contact coverage for probable alcohol use disorders in PRIME implementation areas, 2013–2014.

Characteristic (%, and 95% CI)		Sodo District, Ethiopia			Sehore District, India			Chitwan District, Nepal			Kamuli District, Uganda		
		Total	Men	Women	Total	Men	Women	Total	Men	Women	Total	Men	Women
**AUDIT score of 0 (%)**		26.6 (24.2–29.1)	17.1 (14.2–20.5)	35.9 (32.3–39.6)	86.7 (84.8–88.4)	76.1 (72.9–79.1)	99.4 (98.8–99.6)	71.3 (68.6–73.8)	46.1 (41.7–50.6)	88.2 (85.7–90.3)	85.2 (81.5–88.3)	77.5 (71.8–82.4)	89.9 (85.5–93.0)
**AUDIT positive (%)**		13.9^b^ (12.1–15.9)	25.7^b^ (22.4–29.4)	2.4^b^ (1.5–3.7)	5.6 (4.6–6.7)	10.5 (8.4–12.2)	0.08 (0.01–0.5)	5.0 (3.9–6.4)	11.6 (9.2–14.6)	0.5 (0.2–1.2)	1.7 (1.0–2.8)	4.9 (2.9–8.1)	0.5 (0.1–1.8)
**Dependent alcohol use (%)**		1.8 (1.2–2.7)	3.5 (2.3–5.2)	0.1 (0.04–0.7)	0.7 (0.5–1.1)	1.3 (0.9–2.0)	0.0	0.6 (0.3–1.2)	1.4 (0.7–2.9)	0.0	0.2 (0.04–0.8)	0.5 (0.1–2.4)	0.0
**AUD treatment contact coverage (%)**		13.1^a^^b^ (5.9–26.5)	13.1^a^^b^ (5.9–26.5)	0.0	2.8 (1.2–6.6)	2.8 (1.2–6.6)	0.0	5.1 (1.9–13.0)	5.1 (1.9–13.0)	0.0	3.5 (0.6–19.0)	3.5 (0.6–19.0)	0.0
	Contact coverage from specialist health provider (%)	0.0	0.0	0.0	0.0	0.0	0.0	0.0	0.0	0.0	3.5 (0.6–19.0)	3.5 (0.6–19.0)	0.0
	Contact coverage from generalist health provider (%)	9.1^a^^b^ (3.5–22.0)	9.1^a^^b^ (3.5–22.0)	0.0	1.1 (0.3–4.4)	1.1 (0.3–4.4)	0.0	1.3 (0.3–5.1)	1.3 (0.3–5.1)	0.0	0.0	0.0	0.0
	Contact coverage from complementary provider (%)	3.9^a^^b^ (0.9–15.3)	3.9^a^^b^ (0.9–15.3)	0.0	1.7 (0.6–5.0)	1.7 (0.6–5.0)	0.0	4.5 (1.6–12.4)	4.5 (1.6–12.4)	0.0	0.0	0.0	0.0

^a^ Lifetime treatment contact coverage, rather than over the past 12 months.

^b^ Using an AUDIT cut off score of ≥16.

There were variations in contact coverage for probable depression by provider. Aside from Nepal, the plurality of adults who sought care for probable depression did so from a generalist health provider. In Nepal, 4.0% of adults with probable depression sought care from a complementary provider such as a traditional healer, which exceeded the 3.5% who sought care from a specialist provider and the 1.7% who sought care from a generalist provider.

There were also variations in contact coverage patterns for probable depression by the sex of the affected adult. For example, in India, similar proportions of men and women sought treatment for probable depression (12.3% and 13.1%, respectively). Yet 4.4% of men with probable depression sought help from a specialist provider, compared to 1.8 of women with probable depression. And 3.4% of women with probable depression sought care from a complementary provider, compared to 1.9% of men with depression. A similar pattern is evident in Nepal. In contrast, relatively similar proportions of men and women reported care from the different providers in Ethiopia and Uganda.

The proportion of adults who screened positive for AUD ranged between 1.7% in Uganda to 13.9% in Ethiopia; the proportion of adults whose AUDIT scores were consistent with alcohol dependency ranged from 0.2% in Uganda to 1.8% in Ethiopia. Of the adults with probable AUDs, treatment contact coverage over the past 12 months ranged from 2.8% in India to 5.1% in Nepal. In Ethiopia, lifetime contact coverage for probable AUD was 13.1%. The scarcity of these primary and secondary outcomes complicates our ability to make comparisons by provider or by sex.

## Discussion

We conducted population-based cross-sectional surveys to measure the treatment contact coverage for probable depressive and probable alcohol use disorders in a range of LMIC districts. The screen positive proportions and treatment contact coverage figures varied widely by condition and by country: In PRIME implementation areas, only 8.1% to 23.7% of adults with probable depression and 2.8% to 5.1% of adults with probable AUDs had sought treatment in the past 12 months. These figures generally exceed our hypothesized estimates for baseline treatment contact coverage, yet, they provide an estimate of a substantial unmet need for mental health services. It is the aim of PRIME to meet this need through the implementation of district-level mental health care plans and thus to improve the treatment contact coverage in these populations.

The PRIME Community Surveys advanced on the WMHS findings by focusing on four countries which were not part of the WMHS and by focusing on two disorders for which the tools to close the treatment gap in LMIC are available. Further, the PRIME surveys contribute information to efforts such as the Global Burden of Disease project, which has recognized the scarcity of mental health data available in LMIC [39]. Our findings using the PHQ9 and AUDIT screening tools provide additional evidence from LMIC settings in support of a global pattern of the burden of depression being mostly experienced by women [2] and the burden of AUD mostly experienced by men [3].

Contact coverage figures for probable depression were low (all districts below 24%)—though with some notable variations–and were roughly equitable by sex. Nearly one in four adults in Ethiopia and Uganda who screened positive for depression reported seeking treatment, predominantly from generalist medical providers. Given that neither of these areas had provision for depression care in the public sector at the time of the survey, it is likely that affected participants were seeking help for somatic symptoms (e.g. problems with sleep, appetite or concentration) for which it is unlikely that the underlying disorder would be recognized. However, in these two areas the contact coverage figures can be interpreted as evidence of demand for services. In India and Nepal, community-level sensitization will be required so that affected persons know that they can seek healthcare for their symptoms.

Contact coverage figures for probable AUD (all districts below 14%) were substantially lower than for probable depression. Given the scarcity of women who screened positive for AUD–abstinence appeared to be the norm for women in India, Nepal and Uganda—it is difficult to evaluate the equity of contact coverage by sex. Contact coverage was below 6% in the three areas where contact coverage was measured with 12-month recall, and was 13% in Ethiopia. This result is expected, given the findings from cross-cultural research by Bennett et al; participants in this study felt that medical attention was warranted for serious injuries or loss of consciousness incurred as a consequence of drinking, rather than needing treatment for the drinking itself [40]. As community members and service providers alike are unlikely to believe that a clinical response is warranted for harmful or hazardous drinking behaviours [5], creating and meeting demand for AUD services will require sensitization at the community and facility levels.

The PRIME Community Surveys used screening tools to identify participants who had symptoms consistent with mental disorders. In contrast, the WMHS used the Composite International Diagnostic Interview (CIDI) [29] to assess participants for a range of individual mental disorders. While the CIDI reduces misclassification of disorder status, the use of a diagnostic tool is time- and resource-intensive, and, further, is more challenging for investigators to employ in many LMIC settings, where the majority of untreated people with mental illness are found. Screening tools are advantageous as they facilitate data collection with lay interviewers. Moreover, measurement of mental health disorders with continuous scoring scales allows health system planners to project treatment needs across a range of symptom severity: once mental health services become available, planners can estimate the number of people who will have mild, moderate and severe symptoms, and the likelihood of people from each group to seek treatment.

However, the advantages of using a screening tool to identify potential cases as the denominator of the contact coverage figure must be weighed against the probability of misclassification that is inherent when using a screening tool to identify persons with mental disorders [41]. Subsequent PRIME studies which adapted, translated and validated the PHQ9 against a diagnostic interview revealed that our country-specific cut points had sensitivity and specificity of 61% and 84% in Ethiopia [42], 94% and 80% in Nepal [43], and 15% and 98% in Uganda [44]. Using the prevalence figures we hypothesized in our sample size calculation we can project positive predictive values of between 29% in Ethiopia to 70% in Uganda. For the AUDIT, we made country-specific adaptations as recommend by WHO; yet we are unable to comment on measurement validity of the resulting tools. As such, our figures for the adults who screen positive for depression or for AUD need to be interpreted with caution.

Another important limitation to our study concerns the information available for the population-based sampling design. With few exceptions, limited data are available on the geographic distribution of adults in health service areas of LMIC. As exemplified by the Extended Programme of Immunization sampling methodology [45,46] there are options available to surmount these limitations, and our respective sampling plans demonstrate a range of these options. It is possible that selection bias has affected our results, given the magnitude of replacement participants for adults who were not available or who did not provide informed consent. Specifically, bias in our point estimates (e.g. proportion of screened positive) is possible if these eligible but non-participating adults were systematically different than participating adults with regard to the measurements made in this study. There are other sources of bias worth considering. Social desirability may explain both our high consent rate (all >98%) as well as the contact coverage figures, which often exceeded the 0 to 5% figure used as an input for our sample size calculation. We did not verify the participants self-reported treatment seeking with clinical records. While it is possible that we overestimated contact coverage in this baseline analysis, this bias will be present equally in the follow up survey and so will not affect the planned longitudinal analysis. Further, recall of depression symptoms, alcohol consumption, or treatment seeking over a 12-month period may not be equal through the year, and so any within-country differences in the data collection calendar periods will have to be considered accordingly as part of longitudinal analysis.

There were some noteworthy challenges we faced during the conduct of this cross-country study. In Nepal we found a surprisingly few adults screened positive for depression through routine monitoring during the early data collection phase. A change to the Nepali translation of the term ‘mental health’ to a more locally-relevant term in our informed consent and questionnaire material resulted in a substantial increase in affirmative responses to questions in the PHQ-9 for the last 500 participants in the survey. It is likely that we have underestimated the number of people affected by depression in Nepal. A subsequent validation study in Nepal [43] clarified how detection of depression is improved by including an idiom of distress in conjunction with screening with the PHQ9. In Uganda, due to the substantial distances between the field office and the villages in Kamuli District the field team was not able to schedule interviews during evening or weekend hours. Thus the generalizability of the findings must be limited to a more specific population in the district, namely to those adults who are at home during working hours. Reflecting this, females comprised 66% of the sample in Kamuli, in contrast to 52% of the district’s population as reported in census data [24]. The differential in Chitwan—where we found 60% of the sample was female in comparison to 52% in the census–is most plausibly explained by male out-migration [23,47].

### Future analysis

We plan to conduct a follow-up survey in each implementation area approximately three years after the baseline round. By comparing the baseline vs endline figures, we will be able to determine whether the contact coverage for these disorders has increased–and increased specifically for generalist health providers—following the implementation of the PRIME mental healthcare plans. We will also be able to assess whether contact coverage has increased equitably by sex and by socioeconomic status. In settings where contact coverage has not changed, we will be able to use our Theory of Change framework [48] to systematically evaluate whether the programme implementation reached the required strength and if any element of the mental health care plan did not achieve its hypothesized effect. From the Community Surveys, the assessment of the general health-seeking behaviours and of mental health-related attitudes and beliefs in the implementation areas will inform the final evaluation of the mental health care plans.

## Conclusions

Screening adults for mental health disorders is an important first step in assessing the burden of mental disorders, and then for evaluating the coverage of health services for those disorders. As has been demonstrated here in a range of LMIC, through the use of validated tools and a flexible sampling design, we can confirm the presence of a substantial treatment gap for probable depression and probable alcohol use disorders.

## Supporting Information

S1 Analysis Stata do file for baseline analysis. (TXT)
